# Incorporation mechanism of Fe and Al into bridgmanite in a subducting mid-ocean ridge basalt and its crystal chemistry

**DOI:** 10.1038/s41598-021-00403-6

**Published:** 2021-11-24

**Authors:** Akihiko Nakatsuka, Hiroshi Fukui, Seiji Kamada, Naohisa Hirao, Makio Ohkawa, Kazumasa Sugiyama, Takashi Yoshino

**Affiliations:** 1 Graduate School of Sciences and Technology for Innovation, Yamaguchi University, Ube, 755-8611 Japan; 2 Graduate School of Science, University of Hyogo, Kamigori, 678-1297 Japan; 3 Frontier Research Institute for Interdisciplinary Sciences, Tohoku University, Sendai, 980-8578 Japan; 4 Graduate School of Science, Tohoku University, Sendai, 980-8578 Japan; 5 AD Science Inc., Funabashi, 273-0005 Japan; 6 Present Address: Japan Synchrotron Radiation Research Institute, Sayo, 679-5198 Japan; 7 Graduate School of Advanced Science and Engineering, Hiroshima University, Higashi-Hiroshima, 739-8526 Japan; 8 Institute for Materials Research, Tohoku University, Sendai, 980-8577 Japan; 9 Institute for Planetary Materials, Okayama University, Tottori, 682-0193 Japan

**Keywords:** Mineralogy, Solid-state chemistry

## Abstract

The compositional difference between subducting slabs and their surrounding lower-mantle can yield the difference in incorporation mechanism of Fe and Al into bridgmanite between both regions, which should cause heterogeneity in physical properties and rheology of the lower mantle. However, the precise cation-distribution has not been examined in bridgmanites with Fe- and Al-contents expected in a mid-ocean ridge basalt component of subducting slabs. Here we report on Mg_0.662_Fe_0.338_Si_0.662_Al_0.338_O_3_ bridgmanite single-crystal characterized by a combination of single-crystal X-ray diffraction, synchrotron ^57^Fe-Mössbauer spectroscopy and electron probe microanalysis. We find that the charge-coupled substitution ^A^Mg^2+^  + ^B^Si^4+^  ↔ ^A^Fe^3+^(high-spin) + ^B^Al^3+^ is predominant in the incorporation of Fe and Al into the practically eightfold-coordinated A-site and the sixfold-coordinated B-site in bridgmanite structure. The incorporation of both cations via this substitution enhances the structural distortion due to the tilting of BO_6_ octahedra, yielding the unusual expansion of mean <A–O> bond-length due to flexibility of A–O bonds for the structural distortion, in contrast to mean <B–O> bond-length depending reasonably on the ionic radius effect. Moreover, we imply the phase-transition behavior and the elasticity of bridgmanite in slabs subducting into deeper parts of the lower mantle, in terms of the relative compressibility of AO_12_ (practically AO_8_) and BO_6_ polyhedra.

## Introduction

**Figure 1:**
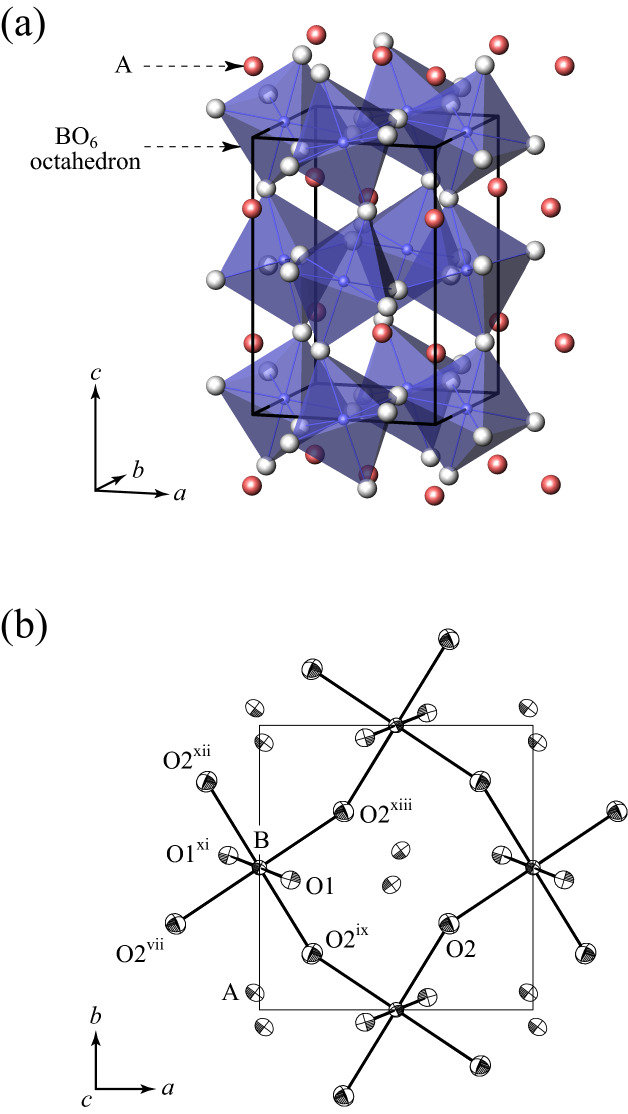
Crystal structure of the present (Fe^3+^, Al)-bearing bridgmanite: (**a**) a view of corner-linked BO_6_ octahedra and (**b**) displacement ellipsoids projected along [001]. In (**b**), atoms are drawn at 80% probability level. Symmetry codes for equivalent atoms are as in Table 5. The software ATOMS (Version 5.1, Shape Software, Kingsport, TN, USA, http://www.shapesoftware.com/00_Website_Homepage/) was used for the crystal-structural representation.

Bridgmanite, with an approximate composition of MgSiO_3_ and the orthorhombic perovskite-type structure (space group *Pbnm*), is believed to be the most dominant constituent of the Earth’s lower mantle. Physical and crystal-structural properties of bridgmanite and their pressure-, temperature- and chemical-dependence provide essential information for detailed understanding of the lower mantle viewed from mineralogical aspects. The crystal structure has the two cation sites, the larger eightfold (nominally 12-fold) coordinated A-site and the smaller sixfold coordinated B-site, consisting of a network of corner-linked BO_6_ octahedra with the A-site atoms located at the centers of cavities in the network and being distorted largely from the ideal cubic structure with *Pm*$\overline{3 }$*m* symmetry owing to the tilting of BO_6_ octahedra (Fig. 1). In the end-member MgSiO_3_ bridgmanite, the A and B sites are occupied only by Mg and Si, respectively.

The incorporation of Fe and Al, important major elements in the mantle composition after Mg and Si, into the two cation sites can affect the physical properties such as the electric conductivity, thermal conductivity, elasticity and rheology of the lower mantle, together with the structural stability of bridgmanite itself. Because of such importance, the effect of Fe and/or Al incorporation on the physical and structural properties of bridgmanite has extensively studied^1–10^. The valence- and spin-states of Fe, its site-distribution, and the creation of cation- and/or oxygen-vacancies depending on these are especially relevant issues associated with the incorporation mechanism of Fe into bridgmanite, because these can strongly influence the electrical conductivity of the lower mantle^1, 9, 11–13^. The creation of oxygen vacancies^14–20^ and the incorporation manner of Fe can also be controlled by the incorporation of Al. In particular, the effect of trivalent Fe^3+^ incorporation is of interest in view of cation partitioning among lower-mantle minerals^21^. The major conclusions on the role of Fe^3+^ and/or Al reported by the previous studies^2, 6, 7, 21–26^ using Fe- and/or Al-bearing bridgmanites are as follows: (i) Fe^3+^ can be distributed preferentially to bridgmanite at lower-mantle pressures; (ii) the presence of Al increases Fe^3+^ contents in bridgmanite; (iii) oxygen vacancies (V_O_) may be created through the substitutions 1/2O^2−^  + ^B^Si^4+^  ↔ 1/2V_O_ + ^B^Fe^3+^ and/or 1/2O^2−^  + ^B^Si^4+^  ↔ 1/2V_O_ + ^B^Al^3+^, where the left superscripts in the chemical formulae represent the occupied sites; (iv) Fe^3+^ can occupy both A and B sites. In particular, Catalli et al.^6^ reported from in-situ synchrotron Mössbauer spectroscopy that both Fe^3+^ and Al are distributed evenly between A and B sites at high pressures, which is accompanied by the high-spin (HS) to low-spin (LS) transition of Fe^3+^. Hummer and Fei^7^ also reported, from Mössbauer spectroscopy, the even site-mixing of both cations in the quenched sample synthesized at 1973–2073 K and 25 GPa. On the other hand, Vanpeteghem et al.^3^ reported from the single-crystal X-ray diffraction study of several (Fe, Al)-bearing bridgmanites with different Fe and Al contents that Fe^3+^ occupies exclusively A-site via the charge-coupled substitution ^A^Mg^2+^  + ^B^Si^4+^  ↔ ^A^Fe^3+^  + ^B^Al^3+^. They furthermore reported that when the Fe content exceeds the Al content, the substitution ^A^Mg^2+^  ↔ ^A^Fe^2+^ occurs for the extra Fe content. Thus, there has been the large discrepancy in incorporation mechanisms of Fe^3+^ and Al between studies. Acquisition of detailed total knowledge of cation distribution, iron valence, and vacancy is necessary for the reliable determination of the incorporation mechanism, but each of the previous studies has not examined them from all of single-crystal X-ray diffraction, Mössbauer spectroscopy and chemical analysis.

Here we characterize the bridgmanite single-crystal with the Fe- and Al-contents expected in a mid-ocean ridge basalt (MORB) component of subducting slabs, by a combination of these three techniques. Bridgmanite formed from the MORB composition contains larger amounts of Fe and Al (~ 0.35 per formula unit for both)^27^ than their amounts (~ 0.05 per formula unit for both)^27^ of bridgmanite expected in a pyrolytic^28^ lower-mantle. This difference in bridgmanite compositions can yield the difference in incorporation mechanism of Fe and Al into the crystal structure between subducting slabs and their surrounding lower-mantle. This should cause heterogeneity in physical properties and rheology of the lower mantle. Elucidating crystal chemistry of bridgmanite formed from the MORB composition is thus a key to solve controversial issues in the lower mantle such as anti-correlated seismic velocity anomalies observed in large low shear velocity provinces (LLSVPs)^10^. From this viewpoint, the precise crystal-chemical examination employing a single crystal is quite significant for detailed understanding of lower-mantle dynamics. In particular, the present study includes the first report on single-crystal structure of bridgmanite with the Fe- and Al-contents expected in MORB. In this paper, we discuss the incorporation mechanism of Fe and Al into bridgmanite in MORB and its structural variation with the incorporation of both cations, and provide earth-scientific implications.

## Methods

### Single-crystal synthesis under high pressure and chemical analyses

Single crystals of bridgmanite were synthesized at 28 GPa and 1873 K using a 5000-ton Kawai-type high-pressure apparatus installed at the Institute for Planetary Materials, Okayama University. The procedure and technique of the experiment are essentially the same as those described in our previous study^29^ as follows. A 10 mm regular octahedron of a sintered MgO containing 5% of Cr_2_O_3_ was employed as a pressure-transmitting medium. The starting materials were the special grade reagents of powdered MgO, SiO_2_, Al_2_O_3_ and Fe_2_O_3_, and mixed in a cation ratio of Mg:Fe:Si:Al = 0.65:0.35:0.65:0.35, which is very close to that^27^ reported for bridgmanite formed from the MORB composition. LaCrO_3_ surrounded by ZrO_2_ thermal insulator was used as a furnace material. The powder mixture was placed in a Pt capsule, which was electrically insulated from the furnace by a MgO spacer. This cell assembly was set in the anvil assembly of tungsten carbide cubes with truncated edge lengths of 3 mm, and then was compressed up to a target pressure of 28 GPa at room temperature. The temperature was then raised to a target temperature of 1873 K at a rate of 100 K/min. The temperature was controlled with a W97%Re3%-W75%Re25% thermocouple, whose junction was put at the midpoint of the outer surface of the Pt capsule. No correction was made for the pressure effect on emf. After being kept under a desired condition (28 GPa, 1873 K) for 2 h, the product was quenched by shutting off the electric power supply. The pressure was released slowly and the product was recovered at ambient condition. Numerical single-crystals of bridgmanite with a size of about 100–200 μm were found in the recovered sample. The color of the crystals is reddish-brown, suggesting the incorporation of Fe ions into the crystals. Compositions of the single crystals (Table 1) were determined by means of a JEOL JXA-8800M electron probe microanalyzer (EPMA). No contamination from the cell assembly materials into the single crystals was detected from qualitative analyses by the EPMA.

**Table 1:** Compositions at five analysis-points (#1–#5) from EPMA and the average of them.

	#1	#2	#3	#4	#5	Average
**Mass% of oxide components**						
MgO	23.34	23.50	23.74	23.46	23.73	23.6(2)
Fe_2_O_3_	24.24	25.00	25.18	24.80	24.68	24.8(4)
SiO_2_	36.31	36.24	34.61	36.15	35.95	35.9(7)
Al_2_O_3_	16.33	16.37	16.61	16.65	16.58	16.5(1)
Total	100.21	101.12	100.14	101.06	100.95	100.7(8)
**Number of cations per O = 3**						
Mg	0.638	0.638	0.654	0.637	0.645	0.642(7)
Fe	0.334	0.343	0.350	0.340	0.339	0.341(6)
Si	0.666	0.660	0.639	0.659	0.656	0.656(10)
Al	0.353	0.352	0.362	0.357	0.357	0.356(4)
Total	1.991	1.993	2.005	1.993	1.997	1.996(14)

### Synchrotron ^57^Fe-Mössbauer spectroscopy

Energy-domain synchrotron ^57^Fe-Mössbauer spectroscopy measurements at room temperature using a nuclear Bragg monochromator were conducted to evaluate Fe^3+^/ΣFe ratio of the present bridgmanite at the BL10XU beamline of SPring-8 (Ref.^30^). The sample was irradiated by the X-ray beam tuned at 14.4 keV from a high heat-load Si(111) double-crystal monochromator. The transmitted X-ray through the sample was monochromatized to around the nuclear resonance energy of ^57^Fe by a high resolution monochromator with a bandpass of about 4 meV, which consists of asymmetric Si(511) and symmetric Si(975) channel-cut crystals. The nuclear monochromator employs a single-line pure nuclear Bragg reflection 333 from an oscillating ^57^FeBO_3_ single-crystal near Néel temperature in the external magnetic field. The bandwidth of the electronically forbidden pure nuclear Bragg reflection was about 15 neV. The source Doppler shift was produced by oscillating the crystal in a sinusoidal velocity mode, which was mounted on a velocity transducer. The absorption spectrum was obtained by counting the intensity of the single-line nuclear Bragg reflection as a function of velocity. The velocity scale was calibrated with respect to a ^57^Fe-enriched standard metallic iron foil with 3 µm thickness under ambient conditions, and the isomer shift was also referenced to the same standard. The spectrum data were collected with a measurement time of 8.3 h. The MossA software package^31^ was used for the computational analysis and the spectrum was fitted using a Lorentzian model. The results are shown in Table 2.

**Table 2:** Comparison of hyperfine parameters from synchrotron Mössbauer spectroscopy with their reference values.

Fe-valence on A/B site	Spin state	IS (mm/s)	QS (mm/s)	FWHM (mm/s)
**The present results**				
–	–	0.40(3)	0.86(4)	0.13(7)
**Reference values**				
^A^Fe^3+^	HS	0.2–0.6^a^	0.7–1.0^b^	–
	LS	− 0.2 to 0.4^a^	1.8–2.4^b^	–
^B^Fe^3+^	HS	0.2–0.6^a^	~ 0.3^b^	–
	LS	− 0.2 to 0.4^a^	1.9–2.9^b^	–
^A^Fe^2+^	HS	0.8–1.5^a^	1.9–2.4^b^	–
	LS	− 0.3 to 0.4^a^	0.8–0.9^b^	–

*IS* isomer shift, *QS* quadrupole splitting, *FWHM* full width at half maximum, *HS* high spin, *LS* low spin; superscripts A and B represents A and B sites, respectively.

^a^Ranges of IS for Fe reported in a variety of compounds^41^.

^b^Ranges of QS for Fe in bridgmanite calculated theoretically^42^.

### Single-crystal X-ray diffraction intensity measurements and structure refinements

The single-crystal X-ray diffraction intensity measurements, data processing and structure refinements were conducted following essentially the same procedures and techniques as those described in our previous studies^32–34^ as follows. The single crystal with a size of 0.10 × 0.08 × 0.04 mm^3^ was selected and then mounted on the tip of a glass fiber for the intensity measurements. The measurements were conducted at room temperature (296 K) using a Rigaku AFC-7R four-circle diffractometer with a graphite-monochromatized Mo*K*α radiation (λ = 0.71069 Å) at an operating condition of 60 kV and 250 mA. The unit-cell parameters were determined by the least-squares method from a set of 25 reflections within the range of 44° ≤ 2θ ≤ 46°. The intensity data of a total of 1774 reflections within 2° ≤ 2θ ≤ 100° were collected using the continuous ω–2θ scan mode and corrected for Lorentz-polarization factors and absorption effects (ψ-scan method). After that, the intensity data were averaged in Laue symmetry *mmm* to give 930 unique reflections. Of these, unique reflections with $\left|{F}_{\mathrm{o}}\right|\le 3{\upsigma } \left(\left|{F}_{\mathrm{o}}\right|\right)$ were eliminated. Even if unique reflections had intensities of $\left|{F}_{\mathrm{o}}\right|>3{\upsigma } \left(\left|{F}_{\mathrm{o}}\right|\right)$ after averaging, those averaged from data set of equivalent reflections including reflection(s) with $\left|{F}_{\mathrm{o}}\right|\le 3{\upsigma } \left(\left|{F}_{\mathrm{o}}\right|\right)$ were also discarded since these reflections were potentially affected by multiple scattering. Moreover, unique reflections with sinθ/λ < 0.26 Å^−1^ were eliminated to reduce secondary extinction effects and to avoid dependence on atomic charge as far as possible in the choice of atomic scattering factors. Finally, 640 unique reflections were used in the present refinements. Internal residuals of the equivalent reflections (*R*_int_) was 0.0131.

The structure refinements were carried out by minimizing the function Σ*w*(*F*_o_ – *F*_c_)^2^ using a full matrix least-squares program RADY^35^. Scattering factors of Mg^2+^, Al^3+^, Si^4+^, Fe^3+^, Fe^2+^ (Table 6.1.1.3 in *International Tables for Crystallography*^36^), and O^2–^ (Tokonami^37^) were used. Anomalous dispersion coefficients for each scattering factor were taken from Table 4.2.6.8 in *International Tables for Crystallography*^36^. Several correction models for the secondary extinction effects were attempted during the refinements, and the isotropic correction of Type II^38, 39^ with a Gaussian particle size distribution model yielded the best fit. The final structure refinement converged smoothly to *R* = 0.0189 and w*R* = 0.0146. The summary of crystallographic data, data-collection and refinement parameters is given in Table 3. The refined structural parameters are given in Table 4. The selected interatomic distances are listed in Table 5. Crystallographic Information File (CIF) is deposited in the Cambridge Structural Database (CSD) (Deposition No. 2089819).

**Table 3:** Summary of crystallographic data, data-collection and refinement parameters.

Chemical formula	Mg_0.662_Fe_0.338_Si_0.662_Al_0.338_O_3_
Temperature (K)	296
Cell setting	Orthorhombic
Space group	*Pbnm*
*a* (Å)	4.8066(4)
*b* (Å)	4.9991(12)
*c* (Å)	7.0233(9)
*V* (Å^3^)	168.76(5)
Crystal size (mm^3^)	0.10 × 0.08 × 0.04
Radiation used	Mo Kα
Diffractometer	Rigaku AFC-7R
Monochromator	Graphite
Scan type	ω–2θ
2θ_max_ (°)	100
Range of *h*, *k*, *l*	0 ≤ *h*, *k* ≤ 10, − 15 ≤ *l* ≤ 15
No. of measured reflections	1774
No. of unique reflections	930
*R*_int_	0.0131
No. of observed unique reflections used in refinements [$\left|{F}_{\mathrm{o}}\right|>3{\upsigma } \left(\left|{F}_{\mathrm{o}}\right|\right)$, $\mathrm{sin\uptheta }/\uplambda \ge 0.26$ Å^−1^]	640
No. of parameters	30
*R*	0.0189
w*R*	0.0146
Weighting scheme	$1/{\upsigma }^{2}\left(\left|{F}_{\mathrm{o}}\right|\right)$

**Table 4:** Refined structural parameters.

Site (W.p.)	A (4*c*)	B (4*b*)	O1 (4*c*)	O2 (8*d*)
Occupancy	0.662 Mg	0.662 Si	1.0	1.0
	0.338(3) Fe	0.338 Al		
*x*	− 0.01684(6)	0	0.11380(17)	0.69272(12)
*y*	0.06041(5)	0.5	0.45654(17)	0.30255(12)
*z*	0.25	0	0.25	0.05895(8)
*U*_eq_ (Å^2^)	0.00649(8)	0.00376(8)	0.00614(18)	0.00697(14)
*U*_11_ (Å^2^)	0.00557(11)	0.00362(10)	0.00614(25)	0.00681(20)
*U*_22_ (Å^2^)	0.00503(12)	0.00354(12)	0.00537(28)	0.00695(20)
*U*_33_ (Å^2^)	0.00889(12)	0.00411(11)	0.00690(27)	0.00714(19)
*U*_12_ (Å^2^)	− 0.00099(9)	− 0.00003(10)	− 0.00025(22)	− 0.00063(17)
*U*_13_ (Å^2^)	0	0.00020(9)	0	0.00077(16)
*U*_23_ (Å^2^)	0	0.00038(8)	0	− 0.00162(17)

*W.p.* Wyckoff position.

**Table 5:** Selected interatomic distances.

Bonds/separations	Distances (Å)
A⋯O1^ii^	2.9159(9)
A⋯O1^i^	3.0834(11)
A⋯O2^iii^, A⋯O2^iv^	3.2292(7)
A–O1^v^	2.0057(9)
A–O1	2.0775(10)
A–O2^vi^, A–O2^v^	2.0437(7)
A–O2^vii^, A–O2^viii^	2.2836(7)
A–O2^ix^, A–O2^x^	2.4884(7)
B–O1, B–O1^xi^	1.8518(3)
B–O2^ix^, B–O2^xii^	1.8213(7)
B–O2^vii^, B–O2^xiii^	1.8241(6)

Symmetry codes for equivalent atoms: (i) *x*, *y* – 1, z; (ii) – *x* – ½, *y* – ½, *z*; (iii) – *x* + 1, – *y*, *z* + ½; (iv) – *x* + 1, – *y*, – *z*; (v) – *x* + ½, *y* – ½, *z*; (vi) – *x* + ½, *y* – ½, – *z* + ½; (vii) *x* – 1, *y*, *z*; (viii) *x* – 1, *y*, – *z* + ½; (ix) *x* – ½, – *y* + ½, – *z*; (x) *x* – ½, – *y* + ½, *z* + ½; (xi)  – *x*, – *y* + 1, – *z*; (xii) – *x* + ½; *y* + ½, *z*; (xiii) – *x* + 1, – *y* + 1, – *z*.

## Results and discussion

### Chemical composition, and valence- and spin-states of Fe

Compositions at five points in a crystal measured by the EPMA and the average of them are shown in Table 1. No significant compositional fluctuation is observed among these five measurement points; this shows that the crystal is almost homogeneous in composition. The averaged composition from the EPMA analyses is calculated as the cation ratio Mg:Fe:Si:Al = 0.642(7):0.341(6):0.656(10):0.356(4) assuming O = 3. This composition shows no significant deviation from the mixing composition of the starting materials in the synthetic experiment, indicating that the crystal includes no significant cation- and/or oxygen-vacancies and is well charge-balanced by trivalent Fe^3+^ ions within the error. Although the preferential occupation of larger Fe^3+^ for A site and of smaller Al^3+^ for B site is inferred in terms of ionic radii^40^ [e.g., HS Fe^3+^  = 0.645 Å and Al^3+^  = 0.535 Å in CN (coordination number) = 6], the degree of their distribution between both sites cannot be inferred from the EPMA data alone.

**Figure 2:**
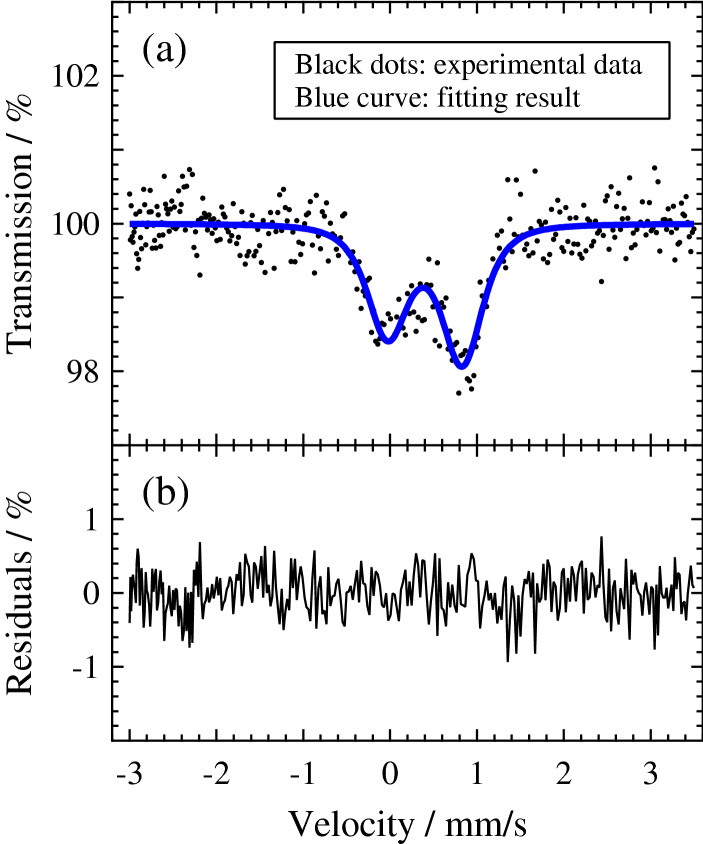
(**a**) Mössbauer spectrum of the present (Fe^3+^, Al)-bearing bridgmanite single-crystal and (**b**) the fitting residuals. The software IGOR Pro (Version 6.2, WaveMetrics, Inc., Lake Oswego, OR, USA, https://www.wavemetrics.com) was used for data graphing.

We here show in Fig. 2a the Mössbauer spectrum of the present bridgmanite single-crystal to gain the more detailed knowledge of valence states, spin states and coordination environments of Fe. The Mössbauer spectrum seems to consist of two absorption peaks with different intensities. The coordination environments around A and B sites (the possible occupied sites of Fe) are largely distorted; this should yield quadrupole splitting, as observed by many researchers^5–7^. The doublets measured using a single crystal can be asymmetric because a certain angle is kept between the principal axis of the electric field gradient tensor in the Fe sites and the incident X-ray beam direction. The Mössbauer spectrum of the present bridgmanite single-crystal should thus be interpreted not as a superposition of singlets but as one asymmetric doublet or a superposition of several asymmetric doublets, depending on the differences in electronic states and coordination environments of Fe. The spectrum is well represented by a Lorentzian model assuming one asymmetric doublet, and the residual peak-components are undetectable from the fitting residuals (Fig. 2b). Models with additional doublets were also attempted, but were not able to significantly improve the fitting quality. The final fit, assuming one asymmetric doublet, gives an isomer shift (IS) of 0.40(3) mm/s and a quadrupole splitting (QS) of 0.86(4) mm/s. These values match well with the reference values^41, 42^ for HS Fe^3+^ on A site and are also close to those for LS Fe^2+^ on A site (Table 2). The latter case is however implausible in terms of the charge balance indicated by the EPMA result. Thus, Fe ions in the present sample exclusively occupy A site in trivalent high-spin state, which leads to that Al^3+^ ions exclusively occupy B site in consideration of the cation ratio indicated by the EPMA result.

To further examine the site distribution of Fe and Al and the presence of vacancies, a preliminary structure-refinement was performed by varying *P*(^A^Fe^3+^), *P*(^O1^O^2−^) and *P*(^O2^O^2−^) as valuable occupancy parameters under the following constraints: *P*(^A^Mg^2+^) = *P*(^B^Si^4+^) ≡ 0.65 (fix), *P*(^B^Fe^3+^) = *P*(^A^Al^3+^) ≡ 0.35 − *P*(^A^Fe^3+^), *P*(^B^Al^3+^) ≡ *P*(^A^Fe^3+^). The resulting *P*(^A^Fe^3+^) was 0.344(2), corresponding to *P*(^B^Fe^3+^) = *P*(^A^Al^3+^) = 0.006. The resulting *P*(^O1^O^2−^) and *P*(^O2^O^2−^) were 1.001(5) and 0.999(4), respectively. Another preliminary structure-refinement was also performed by varying *P*(^A^Fe^3+^) and *P*(^B^Al^3+^) as valuable occupancy parameters under the following constraints: *P*(^A^Mg^2+^) = *P*(^B^Si^4+^) ≡ 0.65 (fix), *P*(^O1^O^2−^) = *P*(^O2^O^2−^) ≡ 1.0 (fix). The resulting *P*(^A^Fe^3+^) and *P*(^B^Al^3+^) were 0.347(2) and 0.356(4), respectively. These show that the mixing of Fe and Al between the two cation-sites and the cation- and oxygen-vacancies are undetectable, agreeing well with the EPMA and Mössbauer results. The final refinement was thus conducted by varying *P*(^A^Fe^3+^) as the only variable occupancy parameter in a model without any cation- or oxygen-vacancies and without any distribution of Fe into B site or Al into A site, under the following constraints: *P*(^A^Mg^2+^) = *P*(^B^Si^4+^) ≡ 1 − *P*(^A^Fe^3+^), *P*(^B^Al^3+^) ≡ *P*(^A^Fe^3+^). The data provided in Tables 3, 4, 5 are from this final refinement. The final *P*(^A^Fe^3+^) is 0.338(3) (Table 4), leading to the cation ratio Mg:Fe:Si:Al = 0.662:0.338:0.662:0.338. This is consistent excellently with the cation ratio from the EPMA.

In ABO_3_ perovskites with the *Pbnm* structure, such as CaTiO_3_ (Ref.^43^), MgSiO_3_ (Ref.^44–46^) and CaGeO_3_ (Ref.^33^), the great structural distortion due to the tilting of BO_6_ octahedra yields much longer separations between an A-site atom and four of twelve O atoms surrounding its A-site atom. As shown in Table 5, in the present bridgmanite, the four longer A⋯O separations, which are not potentially involved in chemical bonding, range between 2.9159(9) Å and 3.2292(7) Å. The remaining eight shorter separations range between 2.0057(9) Å and 2.4884(7) Å; the average of these (2.214 Å) agrees better with the expected A–O bond length (2.25 Å) from HS Fe^3+^ (CN = 8) than the one (2.30 Å) from HS Fe^2+^ (CN = 8), being concordant with the Mössbauer result. The separations between a B-site atom and six O atoms surrounding its B-site atom are close with each other, in contrast to the case of A-site atom, and range between 1.8213(7) Å and 1.8518(3) Å. The average of these (1.832 Å) agrees well with the expected B–O bond length (1.85 Å).

From the consequences of our observations and analyses described above, we conclude that in the present case, in which relatively large amounts of Fe and Al are equally contained, the following charge-coupled substitution is predominant in the incorporation of both cations into bridgmanite:

1 $$^{{\text{A}}} {\text{Mg}}^{{{2} + }} + \, ^{{\text{B}}} {\text{Si}}^{{{4} + }} \leftrightarrow \, ^{{\text{A}}} {\text{Fe}}^{{{3} + }} \left( {{\text{HS}}} \right) \, + \,^{{\text{B}}} {\text{Al}}^{{{3} + }}.$$

Even if there are cation- and/or oxygen-vacancies, divalent Fe2+ ions and mixing of Fe and Al between A and B sites, their amounts/degrees are negligibly small.

### Structural variation with the incorporation of Fe and Al into bridgmanite

**Figure 3:**
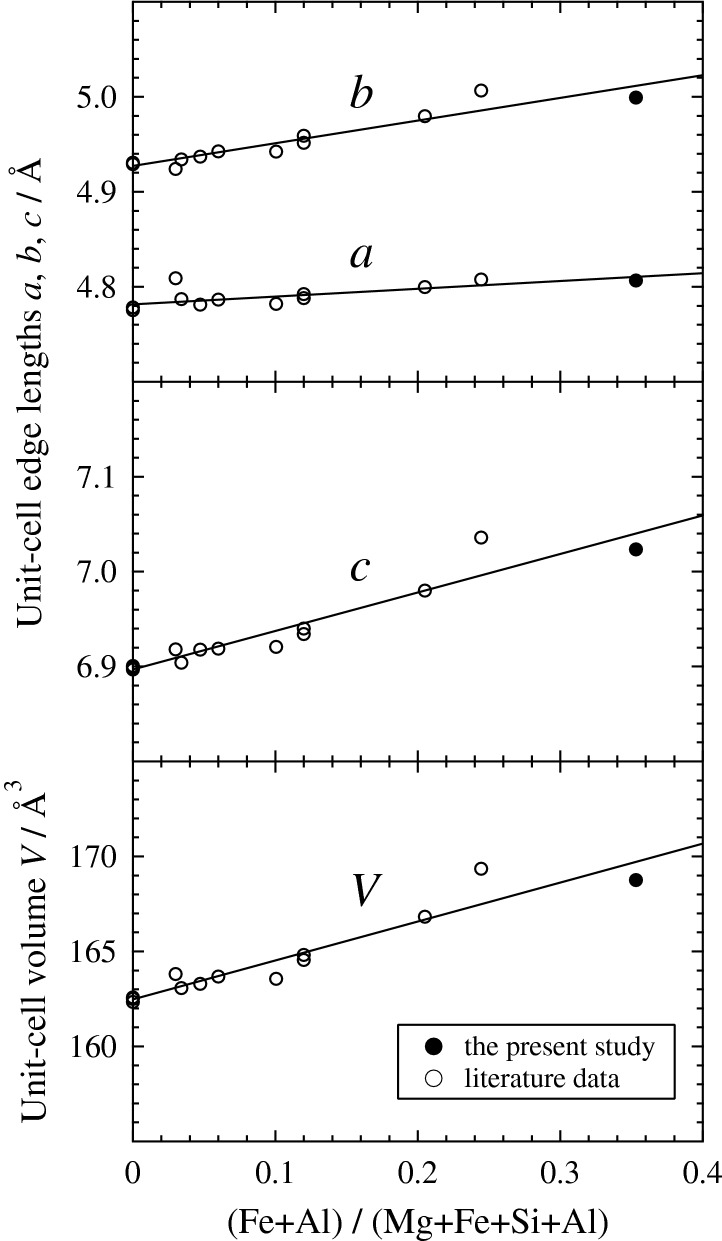
Unit-cell edge lengths (*a*, *b*, *c*) and volume (*V*) as a function of the cation ratio (Fe + Al)/(Mg + Fe + Si + Al). The literature data of the end-member MgSiO_3_ bridgmanite^10, 45, 46, 61^ and the (Fe, Al)-bearing bridgmanites^3, 4, 6, 7, 10^ with near contents between Fe and Al are represented together with the present data. The data graphing software used is as in Fig. 2.

In Fig. 3, the unit-cell edge lengths (*a*, *b*, *c*) and volume (*V*) increase with increasing the ratio (Fe + Al)/(Mg + Fe + Si + Al). This is due to the increase in the mean cation size in the whole of bridgmanite crystal accompanying the incorporation of HS Fe^3+^ and Al^3+^ via the charge-coupled substitution (1) although the mean cation size on A site (<*r*_A_>) decreases by the substitution ^A^Mg^2+^  → ^A^Fe^3+^(HS).

**Figure 4:**
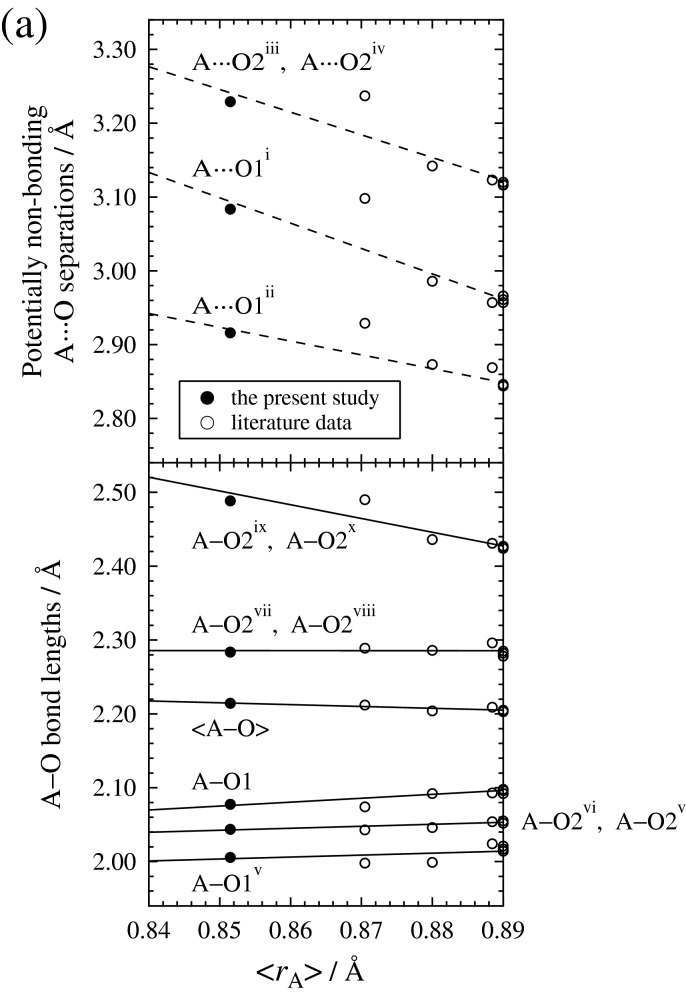

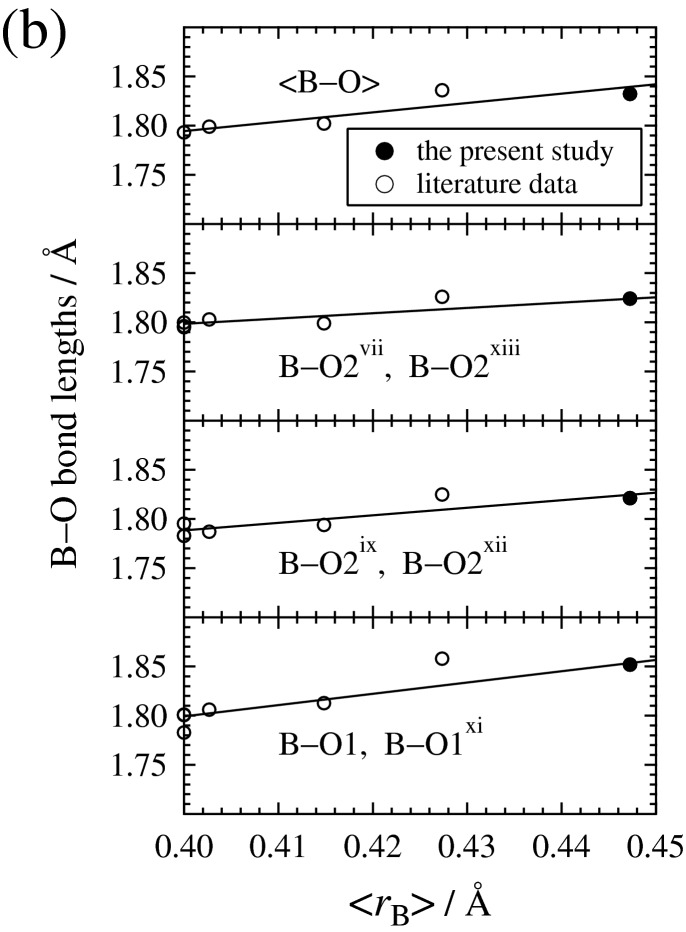
(**a**) A–O bond lengths and potentially non-bonding A⋯O separations as a function of the mean cation size on A site (<*r*_A_>), and (**b**) B–O bond lengths as a function of the mean cation size on B site (<*r*_B_>). The literature data from the single-crystal X-ray diffraction studies of the end-member MgSiO_3_ bridgmanite^44–46^ and the (Fe, Al)-bearing bridgmanites^3^ are represented together with the present data. The < *r*_A_ > values of the samples including the Fe content larger than the Al content were calculated on the basis of the proposal of Vanpeteghem et al.^3^: when the Fe content exceeds the Al content, the charge-coupled substitution ^A^Mg^2+^  + ^B^Si^4+^  ↔ ^A^Fe^3+^  + ^B^Al^3+^ occurs for the Fe content equal to the Al content and the substitution ^A^Mg^2+^  ↔ ^A^Fe^2+^ occurs for the extra Fe content. The data graphing software used is as in Fig. 2.

As observed in Fig. 4a, the increase in <*r*_A_> results in (i) a lengthening of the four shortest A–O bond lengths (A–O1^v^, A–O1, A–O2^vi^, A–O2^v^), (ii) the two almost-unchanged intermediate A–O bond lengths (A–O2^vii^, A–O2^viii^), and (iii) a shortening of the two longest A–O bond lengths (A–O2^ix^, A–O2^x^) and of the four longer potentially non-bonding A⋯O separations (A⋯O1^i^, A⋯O1^ii^, A⋯O2^iii^, A⋯O2^iv^). On the other hand, as observed in Fig. 4b, the increase in the mean cation size on B site (<*r*_B_>) expands all of B–O bond lengths following the ionic radius effect. The expansivities of B–O bond lengths with increasing <*r*_B_> are the largest in the longest B–O1 and B–O1^xi^ bond lengths, running in the direction close to the *c*-axis. This can account for the observation that the expansivities of the unit-cell edge lengths with increasing the ratio (Fe + Al)/(Mg + Fe + Si + Al) are the largest in the *c*-axis length (Fig. 3).

The symmetrical constraints always request 180° for O1–B–O1^xi^, O2^vii^–B–O2^xiii^, and O2^ix^–B–O2^xii^ angles, and the remaining twelve O–B–O angles in a BO_6_ octahedron vary between 87.74(3)° and 92.26(3)°. This shows that the deviation from a regular BO_6_ octahedron is only slightly larger in the present (Fe^3+^, Al)-bearing bridgmanite than in the end-member MgSiO_3_ bridgmanite with the twelve O–B–O angles ranging between 88.49(4)° and 91.51(4)° (Ref.^44^). The incorporation of Fe and Al via the charge-coupled substitution (1), thus, does not largely change the degree of distortion of BO_6_ octahedron and only expands the B–O bond lengths. This shows that the response of the structural distortion to the charge-coupled substitution (1) is dominated mainly by the tilting between corner-linked BO_6_ octahedra as will be described latter. The shortening of the two very weak A–O2 bonds and the four potentially non-bonding A⋯O separations with increasing <*r*_A_> observed in Fig. 4a can be a consequence of a flexible response of these bonds/separations to the structural distortion due to the tilting of octahedra. This brings about the decrease in the averaged A–O bond lengths (<A–O>) with increasing <*r*_A_> , in contradiction to the ionic radius effect.

We here describe the octahedral tilting using the three tilt angles ${\upphi }_{i}^{+}$, ${\upphi }_{i}^{-}$, and ${\upphi }_{i}^{0}$ (*i* = *x*, *y*, or *z*) after Yashima and Ali^43^. The ${\upphi }_{i}^{+}$, ${\upphi }_{i}^{-}$, and ${\upphi }_{i}^{0}$ indicate the in-phase octahedral tilt angle, the out-of-phase octahedral tilt angle, and no octahedral tilting about *i*-axis (*i* = *x*, *y*, or *z*), respectively. The *x*-, *y*-, and *z*-axes represent [100]_0_, [010]_0_, and [001]_0_, respectively, where the subscript “0” represents the pseudo-cubic lattice. The tilting system of *Pbnm* orthorhombic perovskites, represented by bridgmanite, is described by two identical out-of-phase tilting about the [100]_0_ and [010]_0_ axes (${\upphi }_{x}^{-}={\upphi }_{y}^{-}$) and an in-phase tilting about the [001]_0_ axis (${\upphi }_{z}^{+}$). This tilting system is expressed as ${\upphi }_{x}^{-}{\upphi }_{y}^{-}{\upphi }_{z}^{+}$ (${\upphi }_{x}^{-}={\upphi }_{y}^{-}$), corresponding to *a*^–^*a*^–^*c*^+^ in well-known Glazer’s notation^47, 48^. The tilt angles have often been defined only from the fractional coordinates of O atoms^49^, but those will more or less be influenced by the distortions of octahedra themselves. We here calculated the tilt angles via the symmetry-adapted mode approach^50^, which can completely separate the tilts and distortions of octahedra. In terms of this approach, the tilt angle $\upphi$ is given by $\upphi ={\mathrm{tan}}^{-1}\left(2d\mathrm{^{\prime}}\right)$, where $d\mathrm{^{\prime}}$ is the amplitude of octahedral tilt mode. The $d\mathrm{^{\prime}}$ values are converted from the standard supercell-normalized amplitude “*As*” and “*normfactor*” calculated using the program ISODISTORT (or the earlier ISODISPLACE)^51^.

**Figure 5:**
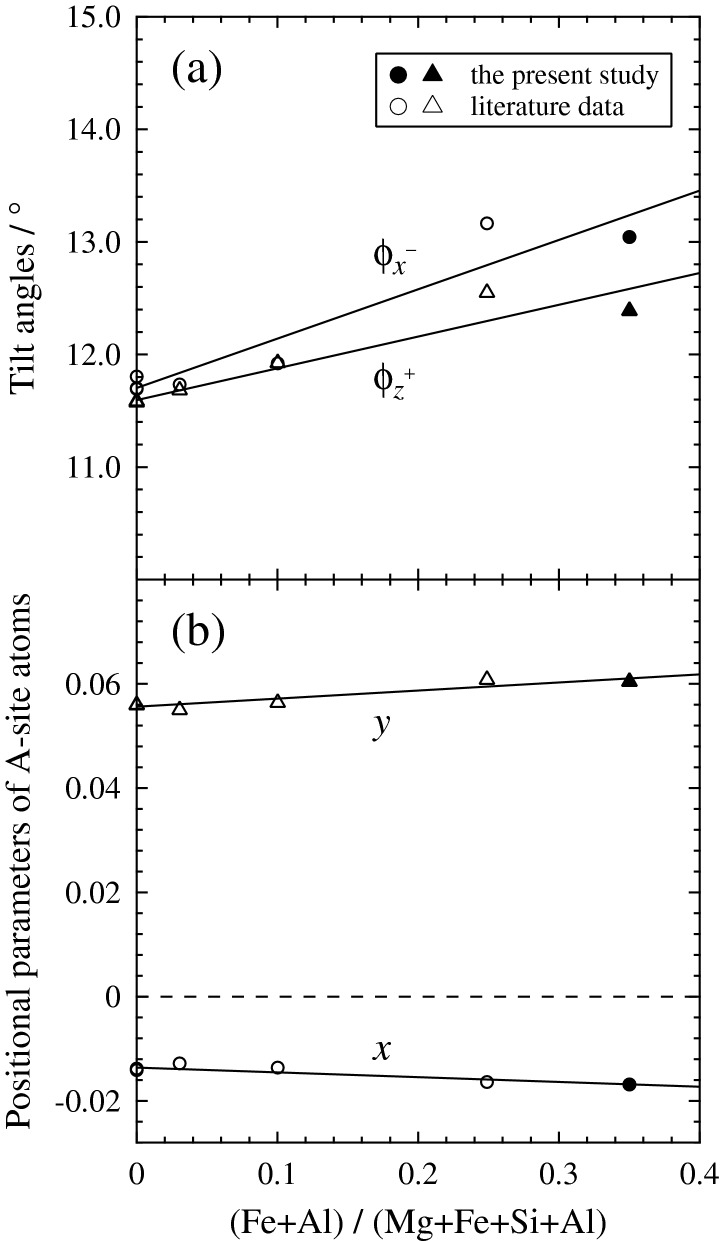
(**a**) Tilt angles ${\upphi }_{x}^{-}$ (= ${\upphi }_{y}^{-}$) and ${\upphi }_{z}^{+}$ of BO_6_ octahedra calculated from the symmetry-adapted mode approach^50^ and (**b**) the positional parameters *x* and *y* of A-site atoms, located at the coordinates (*x*, *y*, 0.25), as a function of the ratio (Fe + Al)/(Mg + Fe + Si + Al). The data calculated from the positional parameters reported in the end-member MgSiO_3_ bridgmanite^44–46^ and the (Fe, Al)-bearing bridgmanites^3^ are plotted together with the present data. The data graphing software used is as in Fig. 2.

The tilt angles ${\upphi }_{x}^{-}$ (= ${\upphi }_{y}^{-}$) and ${\upphi }_{z}^{+}$ of BO_6_ octahedra calculated in this way increase with increasing the ratio (Fe + Al)/(Mg + Fe + Si + Al) (Fig. 5a). The ${\upphi }_{x}^{-}$ and ${\upphi }_{z}^{+}$ are almost equal in the end-member MgSiO_3_ bridgmanite, but the increasing rate of ${\upphi }_{x}^{-}$ with increasing the ratio (Fe + Al)/(Mg + Fe + Si + Al) is larger than that of ${\upphi }_{z}^{+}$. The A-site atom also goes away from its ideal position (0, 0, 0.25), corresponding to the A-site position in the *Pm*$\overline{3 }$*m* cubic structure, with increasing the ratio (Fe + Al)/(Mg + Fe + Si + Al) (Fig. 5b). The structural distortion, i.e. the deviation from the *Pm*$\overline{3 }$*m* cubic structure, is thus getting larger with the incorporation of Fe and Al dominated by the charge-coupled substitution (1). The expansion of the two very weak A–O2 bonds and the four potentially non-bonding A⋯O separations with decreasing <*r*_A_> (Fig. 4a), i.e. with increasing Fe^3+^ content on A site, is a consequence of such increase in structural distortion and a sign of the increased deviation from the *Pm*$\overline{3 }$*m* cubic structure, with twelve equivalent A–O bond lengths and without any octahedral tilting (${\upphi }_{x}^{0}{\upphi }_{y}^{0}{\upphi }_{z}^{0}$).

### Implications for the Earth’s lower mantle

Bridgmanite is now believed to undergo the phase transition to post-perovskite phase with CaIrO_3_ structure, associated with the D′′ seismic discontinuity, at 125 GPa and 2500 K (Ref.^52, 53^). However, some high-pressure studies^54–56^ suggested that another perovskite phase with a different symmetry can intervene between the *Pbnm* perovskite phase and the post-perovskite phase. To examine the possibility of such phase transitions in ABO_3_ perovskites at high pressures and high temperatures, it is effective to discuss the relative compressibility of AO_12_ and BO_6_ polyhedra^57, 58^. The compressibility ratio (β_B_/ β_A_) of the two polyhedra is given by β_B_/β_A_ = *M*_A_/*M*_B_ (Ref.^57^), where the subscripts “A” and “B” represent the AO_12_ and BO_6_ polyhedra, respectively. In *Pbnm* orthorhombic perovskites with largely distorted AO_12_ (practically AO_8_) polyhedra, such as bridgmanite, the parameters *M*_A_ and *M*_B_ are defined as follows:

2 $${M}_{\mathrm{A}}=\left(8{R}_{\mathrm{A}8}/B\right) {\mathrm {exp}} \left[\left({R}_{0}-{R}_{\mathrm{A}8}\right)/B\right]+\left(4{R}_{\mathrm{A}4}/B\right) {\mathrm {exp}} \left[\left({R}_{0}-{R}_{\mathrm{A}4}\right)/B\right]$$

3 $${M}_{\mathrm{B}}=\left({6R}_{\mathrm{B}}/B\right)\mathrm{exp}\left[\left({R}_{0}-{R}_{\mathrm{B}}\right)/B\right]$$

where *R*_A8_, *R*_A4_ and *R*_B_ are the average distances of eight shorter A–O bonds, of four longer A⋯O separations and of six B–O bonds, respectively; *R*_0_ and *B* the bond valence parameters. According to Angel et al.^58^, in perovskites exhibiting zone-boundary type phase transitions, when the BO_6_ octahedra are more rigid than the AO_12_ polyhedra (i.e., *M*_A_/*M*_B_ < 1), the phase transition temperature *T*_c_ rises with increasing pressure as a consequence of the increase in the octahedral tilting; thus, the phase boundary has a positive Clapeyron slope (d*P*/d*T*_c_ > 0). Conversely, when the BO_6_ octahedra are less rigid than the AO_12_ polyhedra (i.e., *M*_A_/*M*_B_ > 1), *T*_c_ reduces with increasing pressure as a consequence of the decrease in the tilting; thus, the phase boundary has a negative Clapeyron slope (d*P*/d*T*_c_ < 0).

The *M*_A_/*M*_B_ ratios at ambient condition, from Eqs. (2) and (3), are calculated to be 0.67 for the end-member MgSiO_3_ bridgmanite^44–46^, 0.72 for the present (Fe^3+^, Al)-bearing bridgmanite, and 0.63 for CaTiO_3_ perovskite^43^ as a good analog of bridgmanite, using the *R*_0_ and *B* values determined by Brown and Altermatt^59^ and the average interatomic distances reported for each compound. It follows therefore that if these A^2+^B^4+^O_3_-type *Pbnm* perovskites undergo phase transitions to perovskite phases with different symmetries at high pressures and high temperatures, their phase boundaries have positive Clapeyron slopes.

The tilt angles of the end-member MgSiO_3_ bridgmanite at ambient condition are calculated to be ${\upphi }_{x}^{-}$ = 11.7° and ${\upphi }_{z}^{+}$ = 11.6° from the reported structural parameters^45^. These values are much larger than those of CaTiO_3_ perovskite (${\upphi }_{x}^{-}$ = 8.3°, ${\upphi }_{z}^{+}$ = 8.8°), which were reported to undergo the phase transitions of *Pbnm* → *I*4/*mcm* at 1512 K and of *I*4/*mcm* → *Pm*$\overline{3 }$*m* at 1635 K under ambient pressure^43^. As mentioned above, when *M*_A_/*M*_B_ < 1, the larger tilting yields the higher *T*_c_, and the rise in pressure further promotes the rise in *T*_c_ because of a positive Clapeyron slope. If the same sequence of the high-temperature phase transitions to higher symmetric L*P* (low pressure)–H*T* (high temperature) phases as CaTiO_3_ perovskite also appears in the end-member MgSiO_3_ bridgmanite at high pressures, thus, *T*_c_ should become much higher than those observed in CaTiO_3_ perovskite at ambient pressure. The incorporation of Fe and Al into bridgmanite would further raise *T*_c_ because it increases the tilt angles ${\upphi }_{x}^{-}$ and ${\upphi }_{z}^{+}$ as shown in Fig. 5a.

In the end-member MgSiO_3_ bridgmanite, Wang et al.^54^ observed the discontinuous changes in the unit-cell parameters and volume at ~ 600 K and 7.3 GPa, suggesting the phase transition to another perovskite phase. This *P*–*T* condition corresponds to a much lower temperature despite a higher pressure than the phase-transition points observed in CaTiO_3_ perovskite. Even if there exists such phase transition in the end-member MgSiO_3_ bridgmanite, therefore, it would be the phase transition not to a higher symmetric L*P*-H*T* phase, such as in CaTiO_3_ perovskite, but to a lower symmetric H*P* (high pressure)–L*T* (low temperature) phase.

At extreme conditions corresponding to deeper parts of the lower mantle, a few in-situ energy-dispersive X-ray diffraction studies using a laser-heated diamond anvil cell have suggested the phase transition of *Pbnm* bridgmanite to another perovskite phase. For example, Meade et al.^55^ reported the phase transition from *Pbnm* (orthorhombic) to *Pm*$\overline{3 }$*m* (cubic) at 64 GPa and 1850 K for (Mg, Fe)SiO_3_ bridgmanite. Shim et al.^56^ reported the phase transition of the end-member MgSiO_3_ bridgmanite from *Pbnm* to one of the three possible symmetries [*P*2_1_/*m* (monoclinic), *Pmmn* (orthorhombic), or *P*4_2_/*nmc* (tetragonal)] above 83 GPa and 1700 K. These suggested space groups after the phase transitions or the presence itself of the phase transitions were not entirely definitive because of low resolution and unreliable peak intensities in the powder X-ray diffraction patterns measured at these extreme conditions. These suggested phase-transition points are however at higher temperatures and higher pressures than those of CaTiO_3_ perovskite, being consistent with the prediction that the larger octahedral tilting and higher pressure raise *T*_c_. The possibility that bridgmanite changes into another perovskite phase with a different symmetry from *Pbnm* before the phase transition to the post-perovskite phase cannot thus be ruled out, whether it is the higher symmetric L*P*-H*T* phase or the lower symmetric H*P*-L*T* phase. Although bridgmanite including high Al and/or Fe contents, such as the present sample (Mg_0.662_Fe_0.338_Si_0.662_Al_0.338_O_3_) with their contents very close to those^27^ reported for bridgmanite formed from the MORB composition, is predicted to have further higher *T*_c_, the phase transition of such bridgmanite to another perovskite phase might be found in slabs that fell/subducted into the lowermost parts of the lower mantle.

The compressibility ratio β_B_/β_A_ of bridgmanite can provide important knowledge of its elastic velocity as well. Comparison of the end-member MgSiO_3_ bridgmanite^45^ and the present (Fe^3+^, Al)-bearing bridgmanite shows that the incorporation of Fe^3+^ and Al through the charge-coupled substitution (1) makes *M*_A_ unchanged, decreases *M*_B_, increases the density ρ, and consequently increases β_B_/β_A_ (= *M*_A_/*M*_B_), where *M*_A_ = 12.87, *M*_B_ = 19.23 and ρ = 4.103 g/cm^3^ for the former; *M*_A_ = 12.92, *M*_B_ = 17.85 and ρ = 4.357 g/cm^3^ for the latter. We can consider that the increase in β_B_/β_A_, depending only on *M*_B_, corresponds to the decrease in the bulk modulus *K*. The bulk sound velocity ${V}_{\mathrm{B}}=\sqrt{K/\uprho }$ is thus expected to decrease with increasing Fe^3+^ and Al contents, which is consistent with the theoretical calculation^60^ for (Fe^3+^, Al)-bearing bridgmanite. This approach from crystallography can thus be a helpful method to gain important insights into the seismic properties within the lower mantle. For this purpose, systematic crystal-chemical studies of bridgmanites with a variety of valence- and spin-states of Fe and compositions are necessary.

## Supplementary Information

Supplementary Information.
