# Supplementary material for: Incorporation mechanism of Fe and Al into bridgmanite in a subducting mid-ocean ridge basalt and its crystal chemistry
Source: Sci Rep. 2021 Nov 24;11:22839. doi: 10.1038/s41598-021-00403-6 (PMC8613292; doi:10.1038/s41598-021-00403-6)

checkCIF/PLATON report

You have not supplied any structure factors. As a result the full set of tests cannot be run.

THIS REPORT IS FOR GUIDANCE ONLY. IF USED AS PART OF A REVIEW PROCEDURE FOR PUBLICATION, IT SHOULD NOT REPLACE THE EXPERTISE OF AN EXPERIENCED CRYSTALLOGRAPHIC REFEREE.

No syntax errors found. CIF dictionary Interpreting this report

Datablock: FeAl34percent_bridgmanite_296K

Bond precision: = 0.0000 A Wavelength=0.71069

Cell:	a=4.8066(4)	b=4.9991(12)	c=7.0233(9)
	alpha=90	beta=90	gamma=90
Temperature:	296 K		

	Calculated	Reported
Volume	168.76(5)	168.76(5)
Space group	P b n m	P b n m
Hall group	-P 2c 2ab	-P 2c 2ab
Moiety formula	Al1.35 Fe1.35 Mg2.65 O12 Si2.65	?
Sum formula	Al1.35 Fe1.35 Mg2.65 O12 Si2.65	Mg0.662 Fe0.338 Si0.662 Al0.338 O3
Mr	442.74	110.69
Dx,g cm-3	4.357	4.357
Z	1	4
Mu (mm-1)	3.956	3.956
F000	217.6	217.6
F000’	218.54	
h,k,lmax	10,10,15	10,10,15
Nref	929	930
Tmin,Tmax	0.691,0.854	0.691,0.854
Tmin’	0.667	

Correction method= # Reported T Limits: Tmin=0.691 Tmax=0.854
AbsCorr = PSI-SCAN

Data completeness= 1.001 Theta(max)= 50.000

R(reflections)= 0.0189(654) wR2(reflections)= wR= 0.0146(640)

S = 1.599 Npar= 30

```
The following ALERTS were generated. Each ALERT has the format
       test-name_ALERT_alert-type_alert-level.
Click on the hyperlinks for more details of the test.
```

Alert level C

```
PLAT041_ALERT_1_C Calc. and Reported SumFormula    Strings  Differ     Please Check
PLAT077_ALERT_4_C Unitcell Contains Non-integer Number of Atoms ..     Please Check
```

Alert level G

```
PLAT004_ALERT_5_G Polymeric Structure Found with Maximum Dimension          3 Info
PLAT005_ALERT_5_G No Embedded Refinement Details Found  in the CIF     Please Do !
PLAT045_ALERT_1_G Calculated and Reported Z Differ by a Factor ...       0.25 Check
PLAT066_ALERT_1_G Predicted and Reported Tmin&Tmax Range Identical          ? Check
PLAT300_ALERT_4_G Atom Site Occupancy of Si         Constrained at      0.662 Check
PLAT300_ALERT_4_G Atom Site Occupancy of Mg         Constrained at      0.662 Check
PLAT300_ALERT_4_G Atom Site Occupancy of Al         Constrained at      0.338 Check
PLAT301_ALERT_3_G Main Residue  Disorder ..............(Resd  1  )        55% Note
PLAT432_ALERT_2_G Short Inter X...Y Contact  Si       ..Si               3.47 Ang.
                                              1/2-x,1/2+y,z  =      6_555 Check
PLAT432_ALERT_2_G Short Inter X...Y Contact  Si       ..Si               3.47 Ang.
                                            -1/2+x,3/2-y,-z  =      2_465 Check
PLAT808_ALERT_5_G No Parseable SHELXL Style Weighting Scheme Found     Please Check
PLAT811_ALERT_5_G No ADDSYM Analysis: Too Many Excluded Atoms ....          ! Info
PLAT882_ALERT_1_G No Datum for _diffrn_reflns_av_unetI/netI ......     Please Do !
PLAT883_ALERT_1_G No Info/Value for _atom_sites_solution_primary .     Please Do !
PLAT966_ALERT_5_G Note: Non-Standard (i.e. 2.0) OMIT Threshold  of        3.0 Sig(I)
```

```
   0 ALERT level A = Most likely a serious problem - resolve or explain
   0 ALERT level B = A potentially serious problem, consider carefully
   2 ALERT level C = Check. Ensure it is not caused by an omission or oversight
  15 ALERT level G = General information/check it is not something unexpected

   5 ALERT type 1 CIF construction/syntax error, inconsistent or missing data
   2 ALERT type 2 Indicator that the structure model may be wrong or deficient
   1 ALERT type 3 Indicator that the structure quality may be low
   4 ALERT type 4 Improvement, methodology, query or suggestion
   5 ALERT type 5 Informative message, check
```

Validation response form

Please find below a validation response form (VRF) that can be filled in and pasted into your CIF.

```
# start Validation Reply Form
_vrf_PLAT041_FeAl34percent_bridgmanite_296K
;
PROBLEM: Calc. and Reported SumFormula    Strings  Differ       Please Check
RESPONSE: ...
```

This compound is a perovskite-type compound with the general formula ABO_3. In the orthorhombic perovskite, such as the present compound, the chemical formula is commonly described as ABO_3 with Z=4 rather than as $A_4B_4O_{12}$ with Z=1. In the present case, A site is occupied by 66.2% Mg and 33.8% Fe and B site is occupied by 66.2% Si and 33.8% Al. Therefore, the present formula is described as $Mg_{0.662}Fe_{0.338}Si_{0.662}Al_{0.338}O_3$ with Z=4 rather than $Mg_{2.65}Fe_{1.35}Si_{2.65}Al_{1.35}O_3$ with Z=1, according to the custom.

```
;
_vrf_PLAT077_FeAl34percent_bridgmanite_296K
;
PROBLEM: Unitcell Contains Non-integer Number of Atoms ..       Please Check
RESPONSE: ...
```

This compound is a perovskite-type compound with the general formula ABO_3, and its chemical formula is $Mg_{0.662}Fe_{0.338}Si_{0.662}Al_{0.338}O_3$. All of Mg and Fe atoms occupy A site and all of Si and Al atoms occupy B site. Both a total of A-site atoms (Mg+Fe) and a total of B-site atoms (Si+Al) are unity. There is no problem because the unit-cell contains integer numbers of A-site atoms and B-site atoms.

```
;
# end Validation Reply Form
```

It is advisable to attempt to resolve as many as possible of the alerts in all categories. Often the minor alerts point to easily fixed oversights, errors and omissions in your CIF or refinement strategy, so attention to these fine details can be worthwhile. In order to resolve some of the more serious problems it may be necessary to carry out additional measurements or structure refinements. However, the purpose of your study may justify the reported deviations and the more serious of these should normally be commented upon in the discussion or experimental section of a paper or in the "special_details" fields of the CIF. checkCIF was carefully designed to identify outliers and unusual parameters, but every test has its limitations and alerts that are not important in a particular case may appear. Conversely, the absence of alerts does not guarantee there are no aspects of the results needing attention. It is up to the individual to critically assess their own results and, if necessary, seek expert advice.

Publication of your CIF in IUCr journals

A basic structural check has been run on your CIF. These basic checks will be run on all CIFs submitted for publication in IUCr journals (*Acta Crystallographica*, *Journal of Applied Crystallography*, *Journal of Synchrotron Radiation*); however, if you intend to submit to *Acta Crystallographica Section C* or *E* or *IUCrData*, you should make sure that full publication checks are run on the final version of your CIF prior to submission.

Publication of your CIF in other journals

Please refer to the *Notes for Authors* of the relevant journal for any special instructions relating to CIF submission.

PLATON version of 03/06/2021; check.def file version of 02/06/2021

Datablock FeAl34percent_bridgmanite_296K - ellipsoid plot

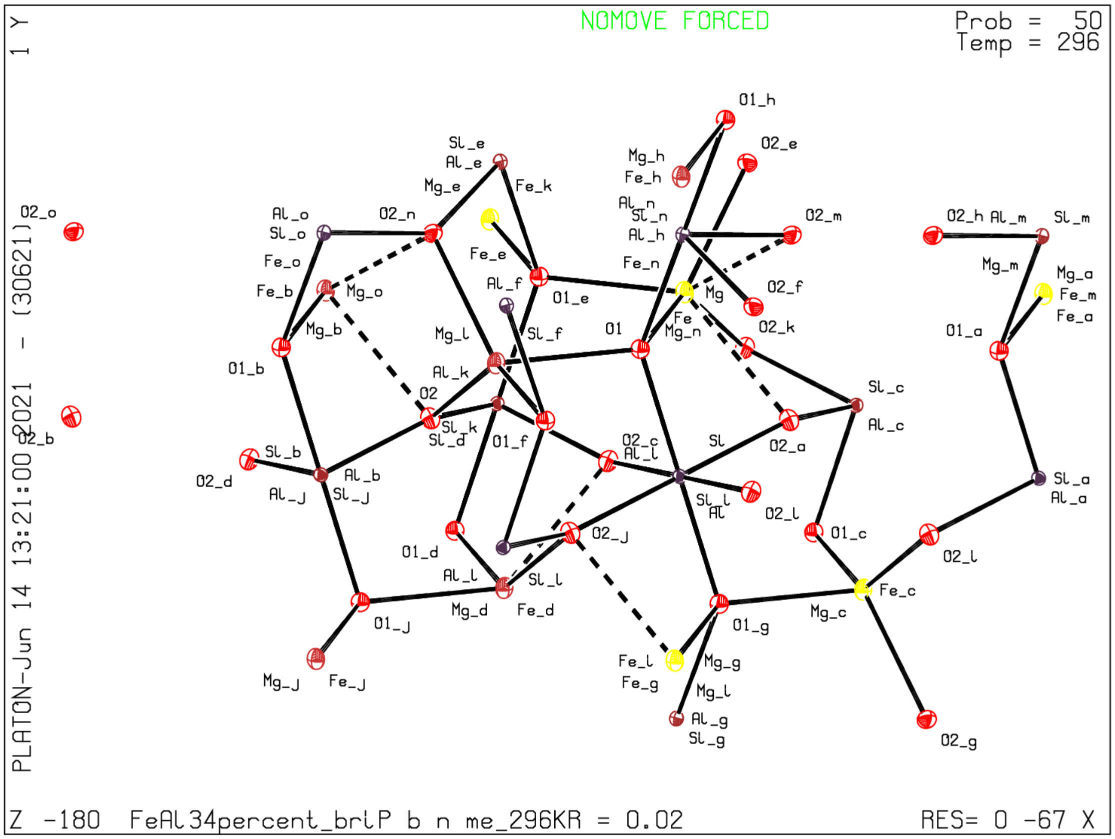

Supplement: Supplementary file 1 — Supplementary Information. [file 41598_2021_403_MOESM1_ESM.pdf]
